# On the Edge of Research and Technological Application: A Critical Review of Electromethanogenesis

**DOI:** 10.3390/ijms18040874

**Published:** 2017-04-20

**Authors:** Ramiro Blasco-Gómez, Pau Batlle-Vilanova, Marianna Villano, Maria Dolors Balaguer, Jesús Colprim, Sebastià Puig

**Affiliations:** 1 Laboratory of Chemical and Environmental Engineering (LEQUIA), Institute of the Environment, University of Girona, Campus Montilivi, Carrer Maria Aurèlia Capmany, 69, E-17003 Girona, Spain; ramiro.blasco@lequia.udg.cat (R.B.-G.); pau.batlle@lequia.udg.cat (P.B.-V.); marilos@lequia.udg.cat (M.D.B.); jesus@lequia.udg.cat (J.C.); 2 Department of Innovation and Technology, FCC Aqualia, Balmes Street, 36, 6th Floor, 08007 Barcelona, Spain; 3 Department of Chemistry, Sapienza University of Rome, P.le Aldo Moro 5, 00185 Rome, Italy; marianna.villano@uniroma1.it

**Keywords:** biocathode, microbial electrolysis cell, methanogenesis, methane, power-to-gas, BES technology, bioelectrochemistry

## Abstract

The conversion of electrical current into methane (electromethanogenesis) by microbes represents one of the most promising applications of bioelectrochemical systems (BES). Electromethanogenesis provides a novel approach to waste treatment, carbon dioxide fixation and renewable energy storage into a chemically stable compound, such as methane. This has become an important area of research since it was first described, attracting different research groups worldwide. Basics of the process such as microorganisms involved and main reactions are now much better understood, and recent advances in BES configuration and electrode materials in lab-scale enhance the interest in this technology. However, there are still some gaps that need to be filled to move towards its application. Side reactions or scaling-up issues are clearly among the main challenges that need to be overcome to its further development. This review summarizes the recent advances made in the field of electromethanogenesis to address the main future challenges and opportunities of this novel process. In addition, the present fundamental knowledge is critically reviewed and some insights are provided to identify potential niche applications and help researchers to overcome current technological boundaries.

## 1. Introduction

In nature methane formation or methanogenesis is accomplished by two main routes: biogenic and abiogenic. Abiogenic methane is produced in much smaller amounts by chemical reactions that do not directly include organic matter. In those cases, methane is produced by either natural thermal splitting of kerogen contained in sedimentary rocks [1] or catalytic formation (Equations (1) and (2), Gibbs free energy values calculated from Thauer et al. [2]) [3,4]. Both are reversible, exothermic and catalyst-dependent reactions. In industry, reaction 1 is used to produce synthetic methane-rich fuel from syngas [5,6], whereas reaction 2 was discovered in the early 1900s by Paul Sabatier and requires elevated temperatures (300–400 °C), high pressure and the presence of a metal catalyst to occur. However, this approach requires large volumes of H_2_ gas externally produced from non-fossil or green sources to be helpful for greenhouse gas mitigation.

(1) $$\mathrm{CO}+{3H}_{2}↔{\mathrm{CH}}_{4}+H_{2}O,\;\Delta G=-151\;\mathrm{kJ}/\mathrm{mol}$$

(2) $${\mathrm{CO}}_{2}+{4H}_{2}↔{\mathrm{CH}}_{4}+{2H}_{2}O,\;\Delta G=-131\;\mathrm{kJ}/\mathrm{mol}$$

Alternatively, biogenic methane formation results from the degradation of organic matter by microbes in anaerobic natural environments, and from different substrates such as CO_2_/H_2_, methanol, formate, methylamines and/or acetate added to engineered bioreactors containing methanogens [7]. Recently, the bioelectrochemical reduction of carbon dioxide (CO_2_) has been postulated as a promising process to obtain methane [8]. In this case, CO_2_ can be either directly reduced by providing electrons as a reducing power (Equation (3)) or by means of the in situ production of hydrogen (H_2_)—an electron donor for hydrogenotrophic methanogenesis—which act as catalyst (Equation (2)) to reduce the large overpotentials affecting the reaction. Indeed, even though the CO_2_ conversion into methane (CH_4_; Equation (3)) theoretically requires a voltage of −0.244 V vs. standard hydrogen electrode (SHE) and pH = 7, more negative values are typically needed.

(3) $${\mathrm{CO}}_{2}+{8H}^{+}+{8e}^{-}↔{\mathrm{CH}}_{4}+{2H}_{2}O$$

### 1.1. Bioelectrochemical Methane Production: Etymology

The term electromethanogenesis was first used to refer to an alternative methanogenic pathway described by Cheng et al. in 2009 [8], where CO_2_ is reduced by a single Archaeon (*Methanobacterium palustre*) using electrical current supplied to the reactor (Equation (3)). Cheng and co-workers observed methane production in a biocathode at a cathodic potential lower than −0.5 V vs. SHE, with 96% of the applied current converted into methane at poised potential of −1 V vs. SHE. Based on the high current densities and the low abiotic hydrogen generation, the fact that methane was directly produced from current rather than hydrogen was suggested for the first time. Although the molecular mechanism of this reaction is still not clear, several studies have suggested that methanogenic archaea can directly take up CO_2_, electrons and protons to produce methane [9,10,11,12]. However, Villano et al. observed that the abiotic hydrogen production was also taking place along with the extracellular electron transfer during the bioelectrochemical methane production. The contribution of these two mechanisms was observed to depend on the set cathode potential. Particularly, the production of molecular hydrogen (Equation (4)), previous to methane production, was observed to enhance the methane yield through the activity of hydrogenotrophic methanogens found in biocathodes [13]. Such claim was supported by other authors, Batlle-Vilanova et al. suggested that only a small amount of methane is directly produced via accepting electrons from the electrode, while most of the production comes from biologically generated hydrogen (Equation (4)) in the biocathode surface followed by hydrogenotrophic methanogenesis [14]. Therefore H_2_ is nowadays considered to be the main electron transfer molecule for bioelectrochemical methane production, especially at low applied cathode potentials [15].

(4) $${2H}^{+}+{2e}^{-}↔H_{2}$$

During the last years, the term electromethanogenesis has been widened to include H_2_-mediated methane production [13,16,17,18,19]. Nevertheless, many authors have employed synonyms in the literature such as “methane biolectrosynthesis” [10], “electromethanosynthesis” [20] or “methane electroautotrophic synthesis” [21]. More recently the concept “bioelectrochemical power-to-gas” [22], encompasses bioelectrochemically produced methane through all catalytic processes taking place using renewable electricity. The objectives of this work are to delimit the concept of electromethanogenesis and clarify the scope of the term, and to assess current state-of-the-art technologies in terms of development of future applications.

The delimitation of new concepts is needed in the field of microbial electrochemistry and microbial electrochemical technologies due to its rapid growth and expansion [23]. To the best of the authors’ knowledge, the term “electromethanogenesis” would be a synonym for “bioelectrochemical methane production”. Up to date, electromethanogenesis has been applied to the process of producing methane using CO_2_ as the sole carbon source, using electroactive microbes in an engineered system (biocathode) powered with electric current. These electroactive microbes were limited to species that can only utilize electrons from a biocathode, however the term should also include both those that accept electrons directly from the cathode and those that accept electrons from another source produced on the electrode. Therefore, it is assumed that electromethanogenesis includes methane formation via (i) direct electron uptake from the electrode (direct electromethanogenesis) and (ii) mediated by hydrogen or other compounds such as formate, acetate or other mediators (mediated electromethanogenesis).

### 1.2. Bioelectrochemical Methane Production: Timeline

**Figure 1:**
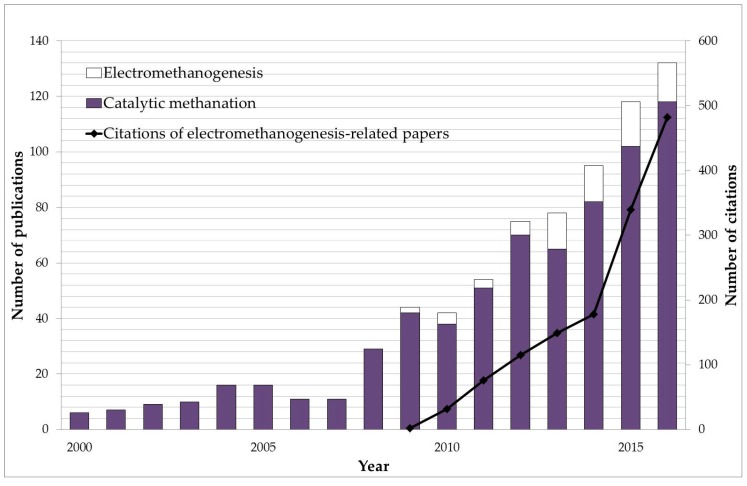
Scientific publications dealing with methane production catalyzed by minerals (catalytic methanation) or microbes (electromethanogenesis) published from 2000 to the beginning of 2017. Additionally, the number of citations of electromethanogesis-related papers is also shown. This data was extracted from Scopus database using the keywords “methane production bioelectrochemical systems”, “electromethanogenesis”, “methane bioelectrosynthesis”, bioelectrochemical methane production”, “electromethanosynthesis”, “methanogenesis bioelectrochemical system” for electromethanogenesis and “catalytic methanation” for catalytic methane formation. (Search date: 4 January 2017).

**Figure 2:**
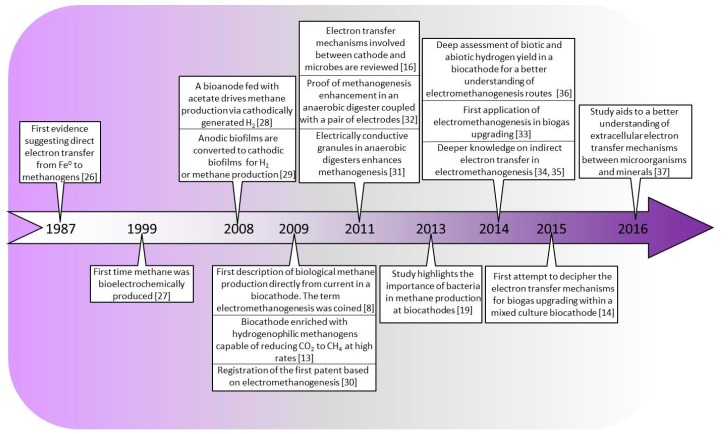
Historical overview of major achievements towards methane production via BESs. In 1987 Daniels et al. reported for the first time the capability of some methanogens to use elemental iron as an electron donor and reduce CO_2_ into CH_4_ [26]. 12 years later, in 1999 Park et al. used a BES with pure and mixed cultures of H_2_-consuming bacteria to produce methane from CO_2_ [27]. They used neutral red as the sole source of reducing power, thus replacing H_2_ as the sole electron donor source. In 2008, works of Clauwaert et al. and Rozendal et al. on H_2_ production at the cathode [28] and the placement of cathodic biofilm for H_2_ and CH_4_ production [29], served as a precursor for the birth of the term [8] just one year later by Cheng et al. During the same year 2009 the first patent based on electromethanogenesis was registered by Cheng et al. [30] and Villano et al. increased the methane production with the enrichment of hydrogenophilic methanogens in the cathodic community [13]. The possible electron transfer mechanisms between microbes and the cathode were reviewed by Rosenbaum et al. in 2011 [16], the same year in which Morita et al. demonstrated the enhancement of electron transfer between methanogens with conductive aggregates in anaerobic digester [31]. Proofs of concept for further application of electromethanogenesis were conducted by Tartakovsky et al. [32] and Xu et al. [33] in 2011 and 2014 respectively, demonstrating methanogenesis enhancement in anaerobic digesters and biogas purification. The importance of the presence of bacteria species in the microbial community to enhance methane production in BESs was highlighted by Van Eerten-jansen [19]. Afterwards, in 2014 Rotaru et al. provided further knowledge of indirect electron transfer routes between microbes for the reduction of CO_2_ into CH_4_ [34,35]. Batlle-Vilanova et al. elucidated the biotic and abiotic hydrogen yield in a biocathode [36], directly linked with the methane yield in electromethanogenic reactors. Electron transfer mechanisms in a bioelectrochemical biogas upgrading process were deciphered in 2015 by Batlle-Vilanova et al. as a first step for further scaling-up of such electromethanogenesis-based technology [14]. Recently, Shi et al. reviewed the extracellular electron transfer mechanisms between microorganisms and minerals, as a basic for designing future methane producing BESs [37].

Most old-standing methane producing technologies attracted the attention of the scientific community before the 2000s. However the scientific interest in catalytic methanation and electromethanogenesis rocketed from 2008 on. As shown in Figure 1, the methanation reaction catalyzed by different metals (i.e., nickel, ruthenium or rhodium)—catalytic methanation—started to be deeply studied from 2008 onwards, most likely due to the boom of anaerobic digestion technology. More recently, the same increasing trend is also followed by manuscripts and citations dealing with bioelectrochemical methane production. This remarkable tendency might be explained by works that appeared between 2007 and 2011, which lead to a better understanding of the relationship between electrodes and microbes (highlighted also in Figure 2). For instance, in 2007 Schröder provided new insight on anodic electron transfer mechanisms [24] expanding the applicability of BES beyond wastewater treatment [25]. Moreover, the first description of the ability of microorganisms to produce methane from CO_2_ reduction by using an electrode in 2009 [8], followed by a better understanding of cathodes as electron donors for microbial metabolism in 2011 [16], may explain why electromethanogenesis-related publications have dramatically increased in the past few years. A recent review on the Bioelectrochemical Power-to-Gas (BEP2G) concept conducted by Geppert et al. is, to the best of our knowledge, the only document of this nature published that has been published thus far [22]. In that review, the performance of methane-producing BESs in relation to cathode potential, electrode materials, operational strategies, and inocula are summarized. On the other hand, this review focuses more thoroughly on the fundamentals of the process, the microbiology and the future niches of the future technology based on electromethanogenesis.

## 2. Electromethanogenesis Pathways, Microbial Communities and Proposed Functionalities

**Figure 3:**
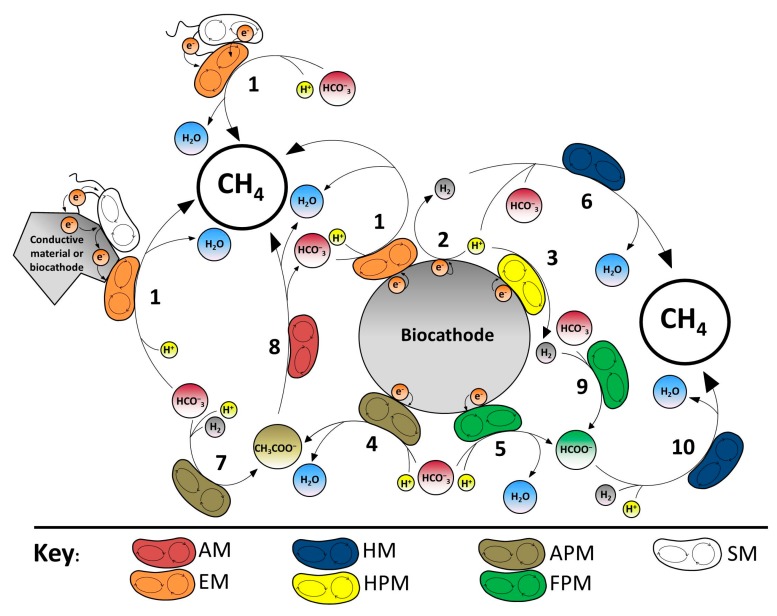
Described electromethanogenesis routes within mixed culture biocathodes. Sizes of the circles do not correspond to any proportions. 1. Direct electromethanogenesis; 2. Abiotic H_2_ production; 3. Biotic H_2_ production; 4. Bioelectrochemical acetate production; 5. Bioelectrochemical formate production; 6. Hydrogenotrophic methanogenesis; 7. Mediated acetate production; 8. Acetoclastic methanogenesis; 9. Mediated formate production; 10. Indirect methane production from formate. AM: Acetoclastic methanogen; EM: Electromethanogen (includes species capable to perform direct and mediated electromethanogenesis); HM: Hydrogenotrophic methanogen; HPM: Hydrogen-producing microorganism; APM: Acetate-producing microorganism; FPM: Formate-producing microorganism; SM: Syntrophic microorganism; Mred: Reduced mediator; Mox: Oxidized mediator.

The three known pathways are taking place in electromethanogenic BESs are (i) CO_2_ reduction (Figure 3: reactions 1, 4, 5, 6, 7 and 9); (ii) methylotrophic; and (iii) acetoclastic pathway (Figure 3: reaction 8). Among them, CO_2_ reduction pathway is considered the major one that drives the methane production, and thus determines the overall performance of the system. However, the importance of the other ones should not be underrated when working with mixed cultures.

Some of the reactions for methane production performed by biocathode communities (Table 1) have been described in the literature, however further research is needed for the elucidation of the molecular pathways involved. Molecular studies are scarce due to their complexity, but a few of them have recently given some insights into how methane is bioelectrochemically produced. For instance, Bretschger et al. correlated electricity consumption with methane production in a mixed methanogenic community suggesting functional correlations between species, especially between members of the family *Desulfovibrionaceae* and the phylum *Euryarcheota* [38]. The high relative abundance in the biocathodes of Desulfovibrionaceae species suggested its involvement in energy transduction (direct electron transfer or hydrogen transfer) between the electrode surface and methanogenic populations. In this study, long-term functional and taxonomic analyses provided new knowledge toward achieving stable and reproducible performance of electromethanogenic reactors. The highest methane production rates occurred when multiple methanogenic phylotypes (*Methanobacterium* sp. *YCM1*, *Methanobacterium bryantii RiH2* and *Methanosarcina mazei Tuc01*) were present in moderate abundance within an electrode-associated microbial community. Moreover, Marshall et al. mapped thirteen genomes from a high-performing electroacetogenic culture and modelled the metabolism of three primary electroacetogens in the community [39].

An overview of possible methanogenic routes within the biocathode compartment according to the literature is shown in Figure 3. The capability of some microorganisms to catalyze the production of hydrogen (Figure 3: reaction 3) and methane (Figure 3: reactions: 1, 6, 8 and 10) with electrons derived from electrodes has been widely studied in BES [8,13,18,40,41]. In addition, methanogens have been found the main microbial community for the reduction of CO_2_ into CH_4_ in BES (Figure 3: reactions 1 and 6) [8,11,13,19,42,43]. Among such community, hydrogenotrophic methanogens (i.e., *Methanobacterium* or *Methanobrevibacter*) have been found to play a main role (Figure 3: reaction 6) [19,44], specifically in studies dealing with mixed culture biocathodes in BESs [45]. Therefore favorable conditions for the growth and the activity of hydrogenotrophic methanogens, such as better bioavailability of H_2_, might lead to an increase in methane production by these systems.

Bacteria species might also have an important role in methane production (Figure 3: reaction 1 with syntrophic microorganisms involved) [46]. Furthermore, a current network based on microbial nanowires as electric connectors has been described between bacteria and methanogens in methane production environments (Figure 3: reaction 1 with syntrophic microorganisms connected to methanogens via nanowires) [31]. Results from studies analyzing microbial communities may lead to hypothesize that bacteria enhance methane production not only by catalyzing hydrogen production (i.e., *Hydrogenophaga caeni* and *Desulfovibrio putealis*) as an intermediate for methane generation [45], but also by consuming oxygen (i.e., *Hydrogenophaga caeni*, *Methylocystis* sp. and *Acidovorax caeni*), which causes toxicity to methanogenic archaea [11,19].

Moreover, some microbes are able to use cathodic electrons to produce reduced compounds such as hydrogen (Figure 3: reaction 6) or formate (Figure 3: reaction 10) at rates exceeding their own metabolic capacity to use them [47]. Consequently, these compounds become available as substrates to other hydrogenotrophic strains and could potentially sustain a diverse electromethanogenic cathodic biofilm.

### Electron Transfer from Cathode to Microbes in Electromethanogenesis

**Figure 4:**
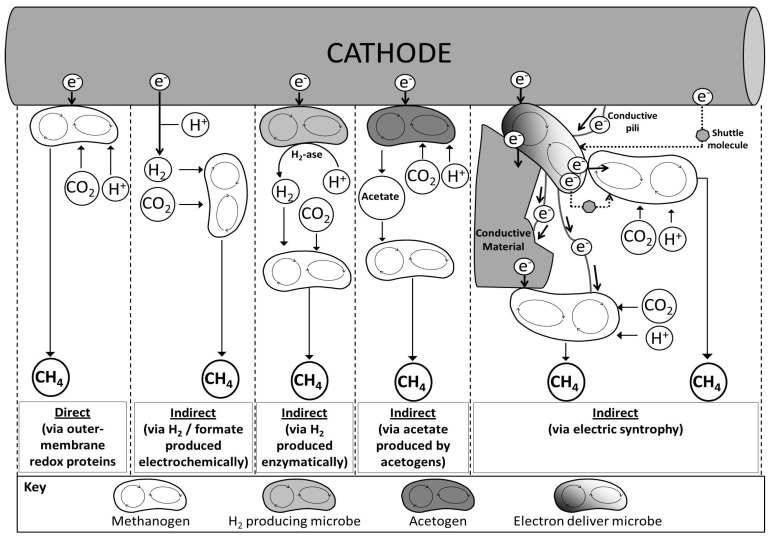
Proposed electron transfer mechanisms within the biocathode compartment. Even though H_2_ is the only intermediate molecule (bio)electrochemically produced on the surface of the electrode shown in the figure, formate or other molecules must be also considered to play the same role as electron carrier. Sizes of the circles do not correspond to any proportions.

Energy supplied in electromethanogenesis is mediated by electric current flowing through conductive solid materials (electrode) and/or small molecules acting as energy carriers (Figure 4). From the electron transfer mechanism perspective, methane production in a biocathode aimed for electromethanogenesis can take place directly and/or indirectly. Similarly to a bioanode, direct electromethanogenesis would take place in the biocathode through outer membrane redox proteins which are in contact with the electrode such as *c*-type cytochromes [82,83], ferredoxin, rubredoxin, hydrogenase and/or formate dehydrogenase [84], in addition to electrically conductive pili (nanowires) [85]. Mediated electromethanogenesis could also take place by using (i) electrochemically or bioelectrochemically produced H_2_ [13,14]; (ii) formate [71] or biologically produced acetate [42,55,64,65,66,67,68,69,70]; (iii) soluble external mediators such as flavins [86], riboflavins [87], quinones [88] and phenazines [89] secreted by microbes or not (i.e., humic acids, thionine, viologens, methylene blue, and sulfur species) [90,91]; and/or (iv) interspecies electron transfer (IET) [34,35] through similar electron carriers, nanowires [87,92] or *c*-type cytochromes as membrane-bound proteins [82,93,94,95].

Recent studies have given some insights into how microbes interact with cathodes [16,84], where hydrogenotrophic methanogens were considered crucial players in the process [48,73,96]. Controversy appeared when some studies questioned the direct electron uptake from electrodes, in which electromethanogenesis mechanism was first based. In 1999, Park et al. were the first to report the ability of a methanogenic mixed culture to drive methanogenesis with an electrical current using neutral red as electron shuttle [27], according to the given terminology this would be considered mediated electromethanogenesis. Afterwards, Cheng et al. [8] found electromethanogenesis without the use of an artificial mediator, suggesting the primacy of a direct electric pathway over a mediated electromethanogenesis in a culture dominated by *Methanobacterium palustre*. In another study, Villano et al. [13] demonstrated that only a fraction of methane was produced by direct electromethanogenesis with the remainder being generated via H_2_-mediated electromethanogenesis by hydrogenotrophic methanogens present in the bioelectrochemical reactor and the prevalence of a mechanism over the other on the overall methane production was highly dependent on the potential applied to the biocathode. Moreover, Deutzmann and colleagues attributed extra product to extracellular redox-active enzymes sorbed to a redox-active surface catalyzing the formation of compounds (e.g., H_2_ or formate), which function as electron donors in microbial catabolism [50]. The authors also claimed that redox-active enzymes, such as hydrogenases and formate dehydrogenase, can be present in cell-free spent medium and interact with a cathodic surface [50].

On the other hand, evidence of direct electron transfer mechanisms suggested that hydrogenotrophic methanogen *Methanosarcina* sp. and acetoclastic methanogen *Methanosaeta* sp. can produce methane via CO_2_ reduction by exploiting direct electron transfer from elemental iron [26,49,51], iron oxides [97], activated carbon [98] and other microorganisms [34]. Recently, *Methanobacterium*-like strain IM1 was also found to be capable of direct electromethanogenesis [52] at a cathode potential of −0.4 V versus SHE, at which a hydrogenotrophic control strain (*M. maripaludis*) did not produce methane. In addition, another study conducted by Lohner et al. provided direct evidence of the capability of hydrogenotrophic methanogens to accept electrons directly from a cathode using a hydrogenase-disrupted mutant of *Methanococcus maripaludis* [48]. In the study they suggested that even though a direct hydrogen-independent pathway for extracellular electron transfer exists, most of the electrons used for methanogenesis were indirectly derived from hydrogen. To elucidate the electron transfer mechanisms within direct electromethanogenesis, future research should focus on the role of extracellular enzymes in the process, taking into account that different species might carry out different electron transfer strategies, thus explaining such contradictory findings [99].

In addition, the cooperation of multiple microbial species in terms of energy exchange, known as electric syntrophy [100] or IET [101], has been also described in electromethanogenic biocathodes [102]. Electron exchange via IET is similar to electron uptake from the cathode. In order to elucidate syntrophic cooperation between species, co-culture studies representative of such interactions have been conducted, suggesting that indeed IET can occur between bacteria and methanogens in electromethanogenesis [102]. IET between species such as *Geobacter metallireducens* and electron-accepting methanogens of the *Methanosaeta* and *Methanosarcina* genera to reduce CO_2_ into CH_4_ was observed by Rotaru et al., in spite of the acetoclastic nature of both methanogens [34,35]. *Methanosaeta harundinacea*, another methanogen, can also interact with bacteria *G. metallireducens* in a co-culture by using additional electrons from ethanol fermentation to reduce CO_2_ into CH_4_ via IET [34]. In any case, syntrophic interactions within methanogenic conditions are highly dependent on the organisms present [34], and the presence of high conductive materials [97,98,103,104,105]. In fact, enhancements of methanogenesis through electric syntrophy have been reported in studies using conductive materials such iron oxides [106,107], graphite [98,108], biochar [103], and microbial nanowires [31,35]. Indeed, many methanogens have shown inability to directly uptake electrons from the cathode, thus being dependent from other species such as *Geobacter* [31,109,110,111,112] or *Acetobacterium* species that function as electron bridges between methanogens and the cathode [47].

Nevertheless, still a deeper understanding of the biochemical energetics of the reaction mechanisms is necessary to optimize electromethanogenesis. In this sense, Rosenbaum et al. suggested that energy gain for biocathode application must be maximized, while microbial energy consumption minimized [16]. According to the authors, the microbes energy gain from biocathodic reaction is strongly affected by the type and efficiency of the extracellular electron transfer mechanism utilized. Yet, supplementary energy must be added in different forms (e.g., light, organics) to maintain biocatalytic activity when microbes are not capable to conserve energy during the biocathodic reaction. Undoubtedly, improved designs in electromethanogenic BESs must walk hand in hand with the elucidation of the electric routes. A better understanding of electrode-microbe and microbe-microbe interactions is required in order to increase the efficiency of future electromethanogenic BESs. A possible approach to increase that knowledge might be the use of molecular techniques to elucidate molecular mechanisms. In this sense, an study conducted by Sydow et al., in which all the current molecular techniques able to be used in the elucidation of electron transfer mechanisms of electroactive species were gathered, are of high interest [113]. In this sense, genome sequencing, conjugation, gene disruption, electroporation and the use of suicide/natural plasmids and gene promoters have become extremely valuable in bioelectrochemistry. Recently, many reviews in the literature have focused not only on the electrode-microbe [40,114] and microbe-microbe interactions [115,116], but also on the biotechnological potential of such an electrical network [64,101,117,118,119,120]. Moreover, reviews on electroactive microorganisms themselves [121,122] and their capability to transfer electrons through conductive materials or pili in prokaryotic organisms [123], enabling electrical exchange, have recently appeared in the literature as a result of an increasing attention by the scientific community.

## 3. Applications of Electromethanogenesis

The niche of a future technology based on methane bioelectrochemically produced from CO_2_ is not clear yet. However, electromethanogenesis-based technologies have a great potential for storing renewable energy in the form of methane, improving waste treatment processes or upgrading gas streams containing CO_2_. In all cases, future studies must focus on further up-scaling, increase process efficiencies and reduce operation costs in order to reach coexistence with well-established technologies, or even a hypothetical overtaking. Those improvements will be achieved by targeting the current limitations of the process—later discussed in this review—such as side reactions, mass transport, inoculum type, electrode material, membranes, operation parameters, up-scaling, and the lack of novel efficient reactor designs. Although these constraints are common to all BES processes, the authors think that, what is specifically referred to CH_4_-producing BES, the establishment of the best conditions, which allow the growth of a well-stablished methanogenic biofilm, are crucial. However, since these biofilms are dependent on several parameters such as the cathode potential, electrode material, pH, etc. only an extensive knowledge of how they interact between themselves will permit electromethanogenesis to be the basis of a competitive technology.

### 3.1. Renewable Energy Storage: The Bioelectrochemical Power-to-Methane Concept

Electromethanogenesis provides a new way to store electrical energy in a stable form as “carbon-neutral” methane [18,42,124], becoming an attractive technology for bio-methane production and renewable energy storage integration [18,22,125,126,127]. Furthermore, the process enhances the role of BESs within the waste biorefinery concept for the production of biofuels and chemicals [128,129,130]. High efficiencies and yields of electromethanogenesis-based technologies will allow for a future applicability of BES technology for energy storage in the stable form of biomethane, which could be also directly injected into the existing gas grid, or used as vehicle fuel [131]. This technology will become really interesting during events of intermittent renewables electricity from solar and wind [124], whose surpluses are nowadays unsuccessfully recovered. The stored methane could be converted back into energy and electricity when needed [132]. In this sense, Sato et al. also proposed to apply electric energy in depleted oil fields re-filled with CO_2_, in which electroactive microbes would perform electromethanogenesis [9]. Besides its feasibility as an energy carrier [133,134,135], methane could also be applied as a precursor for green fuels such as biodiesel, methanol and other hydrocarbons [136].

Bioelectrochemical methane production from renewable energy is nowadays considered the main application of electromethanogenesis. In terms of productivity, the highest electromethanogenic-BES methane yield reported so far is 2.9 mol CH_4_ day^−1^ m^−2^ at a potential of −1.2 V (vs. SHE)—lowest potential of the assessed studies—using a hybrid graphite felt biocathode [20]. Table 2 gives an overview of the operational parameters (in terms of both reactor operation and cathode material) and performance of methane-producing biocathodes so far. Both methane production rate and cathode capture efficiency (CCE) have been considered to evaluate the process performance, with CCE being a key parameter representing the fraction of equivalents of current recovered as methane.

### 3.2. Electromethanogenesis for Biogas Upgrading

Many efforts are being made to improve the typical CH_4_:CO_2_ ratio of around 3:2 in the biogas in order to improve the quality of the product for novel applications, such as biomethane. Among the existing biological biogas upgrading technologies, BES-based technology has recently emerged as a promising alternative [33]. A proof of concept carried out by Xu et al. and it resulted in an enriched biogas—CO_2_ content <10% (*v*/*v*)—with a better performance of the upgrading process when the electrodes were placed within the anaerobic digester and the system was operated in continuous mode. Moreover, several recent studies also showed that the integration of anaerobic digestion (AD) and BES may accomplish biogas upgrading [142]. For instance, Bo et al. inserted an electrode in an AD reactor increasing the content of methane up to 98% (*v*/*v*), mainly due to the in situ reduction of CO_2_ by the hydrogenotrophic methanogens which used the H_2_ produced in the electrode as an electron donor [143]. Other studies contributed to deepen knowledge of the electron transfer mechanisms for biogas upgrading to biomethane within a mixed culture biocathode, also reporting hydrogenotrophic methanogenesis by Methanobacterium sp. as the main mechanism for methane production in the biocathode rather than direct electromethanogenesis [14]. Regardless of the lack of long-term operation studies in larger digesters, the promising results showed in lab-scale demonstrate the need to focus the efforts in a further up-scaling of the process.

### 3.3. Electromethanogenesis Coupled to Waste Treatment

Electromethanogenesis has recently been considered at the service of waste treatment technologies. Among them, AD technology, as it enhances methane production increasing the overall efficiency of the process. Several works have reported an increase of the methane yield and/or a better decomposition of complex substrates when coupling BES to anaerobic digesters [31,32,72,80,109,144,145,146,147,148,149,150,151,152,153,154,155,156,157,158]. However, de Vrieze et al. attributed those improvements to an increase of biomass retention on the electrodes, rather than electrochemical interaction [159]. Nevertheless, electromethanogenesis has been reported to take place in psicrophilic [145], mesophilic [8,13,146,160], and thermophilic conditions [15,53,161] proving robustness to the technology. Besides the introduction of electrodes directly in the anaerobic reactor, methane production could be enhanced by the addition of carbon-based (i.e., carbon cloth, carbon felt, and granular activated carbon) [76,147,162] or non-carbon-based [163] conductive materials as a method to increase electro-active methanogenic activity. Moreover, the coupling of an external cathode to the anaerobic digester resulted in an improvement of its performance [148]. Bo et al. reported a CH_4_ yields, chemical oxygen demand (COD) removal rates and carbon recovery rates that were 24–230%, 130–300% and 55–56% higher than traditional AD reactors [143]. Likewise, the authors showed an enhancement of the activities of enzymes associated with hydrolysis-acidification, together with a decrease of oxidative-reductive potential when an iron anode and a graphite cathode were inserted into AD for waste activated sludge treatment. Song et al. also proved that the electromethanogenesis coupled to anaerobic digestion system could obtain much higher volatile solids reduction and energy recovery with shorter hydraulic retention times than the conventional AD technologies [152]. In addition, Feng et al. experienced a boost in the growth of methanogens, a higher removal of suspended and volatile suspended solids and an increase of CH_4_ production by 22.4% when the anode served as a source of electrons by self-oxidation and release of Fe^2+^ in an iron-graphite electrode placed in an anaerobic reactor [164]. Moreover, the introduction of the anode and cathode in an upflow anaerobic sludge blanket, together with the addition of zero valent iron, raised the COD removal from 60% to 95% and increased reactor stability in another study [155].

Four possible mechanisms have been proposed for electrochemical enhancement of the AD: (i) the release of anodic oxygen formed during water electrolysis in the AD broth leading to a microaerophilic environment and a boost of substrate hydrolysis [165,166]; (ii) cathodic hydrogen formation by the electrolyzer as a co-substrate for the fermentation with enhanced methane formation by hydrogenotrophic methanogenesis; (iii) direct electron transfer to methanogens attached to the cathode surface [8]; and (iv) the enhancement of the biomass retention on the electrodes attached inside the AD reactor [54,74,159]. However, the second mechanism proposed highly depends on the reaction taking place in the counter electrode—usually water electrolysis and/or oxidation of organic matter*—*which, in turn, depends on the potential applied and the electrode material.

In addition, syntrophic reactions may have an important role on interspecies electron transfer in the digester. Related findings suggest that IET may alter the conventional concept of ATP generation and could be an important mechanism for hydrogenotrophic methanogenesis in AD [31].

Early studies suggested that direct electromethanogenesis holds some potential advantages compared to traditional anaerobic digestion processes [167]. First, in the case of a two chambered BES, organic matter oxidation and methane production are two physically separated processes occurring at the anode and the cathode, respectively. This allows protecting methanogens at the cathode against inhibitory compounds contained in the waste stream which is treated at the anode, as well as, increasing the methane content in the produced biogas. Second, electromethanogenesis is more energetically feasible since it can occur at ambient temperature or, if operated at higher temperatures (e.g., 35–55 °C), still there is no need for heating the influent waste stream (separately treated at the anode). Third, BESs can treat waste streams with low organic matter content, where AD cannot take place [140].

In a typical electromethanogenesis-based BES, hydrogen gas produced by the cathode can act as an electron carrier for hydrogenotrophic methanogenesis. As hydrogen gas is only measured in the gas phase, this process cannot be quantitatively measured yet. Thus, finding a sensor to quantify methanogenesis via hydrogenotrophic archaea may lead to a better understanding of electromethanogenesis and its further scaling up. In most cases none or very little hydrogen is measured in the gas phase, which may indicate either immediate conversion to methane or that hydrogen gas is not produced at all [54,74,155]. Nevertheless, new approaches of how to couple more effectively both technologies are needed. Indeed, some authors are already working on developing new reactor designs to exploit the existing synergies between the electromethanogenesis and AD with promising results [168].

In addition to the application of electromethanogenesis in AD, the potential of CH_4_-producing BES on treating medium/low strength wastewater while maximizing energy recovery from organic substrates attracted the attention for its application on wastewater treatment (WWT) [138,169]. Wastewater as an electron donor is desirable due to its low price, availability, and the growing awareness for ecological WWT with a minimum carbon output. Bacteria in these systems degrade organic material in the anode, and transfer electrons to the cathode electrode. Even if organic matter is used as electron donor, an additional voltage is supplied to BESs to overcome thermodynamic limitations and produce H_2_ or CH_4_ in the cathode compartment [28,170,171,172]. WWT in BESs has potential benefits compared to traditional AD. For instance, the physical isolation of the waste organic compounds oxidation phase from the methane generation enables (i) the protection of the methanogenic culture against inhibitory substances which may be present in waste streams and (ii) to generate biogas with higher methane content, and less CO_2_ and other impurities. WWT with BESs must face several important challenges to achieve practical implementation at large scale [25]. The former demand of using real wastewater in the studies has been fulfilled and resulted in a better understanding of how to operate such systems. In many cases, the use of real wastewater [139,144,168,173,174,175,176], sewage sludge [152,153], waste activated sludge [177], digested or non-digested slurry [178] was supplemented by other substrates such as glucose [168] or sodium acetate [144] to enhance microorganism growth. Nevertheless, further studies are required to develop strategies for improving the degradation of complex materials and controlling the microbial reactions occurring in the system [25]. Zeppilli et al. fed simulated municipal wastewater in the anode of a BES and experienced a stable and robust performance in terms of current generation (coulombic efficiency, CE, higher than 70%), and COD removal (70% on average), whereas methane production took place in the cathode [56]. However, the overall performance was lower than that obtained by feeding the anode with acetate as sole electron donor, due to the presence of slowly biodegradable COD in the municipal wastewater-like feeding.

In the broader context of both biogas upgrading and wastewater treatment, particularly attractive is the possibility to integrate the anaerobic digestion technology with a CH_4_-producing BES. Indeed, the AD liquid effluent generally consists of diluted organic acids which can be further removed at the anode of the bioelectrochemical system whereas the carbon dioxide contained in the AD biogas can be converted into additional methane at the cathode. Also, by using a cation exchange membrane to separate the anode and cathode compartments in the BES, this integration opportunity has been proposed as a strategy to remove ammonium, along with organic substrates, from the AD liquid effluent [138].

However, new designs for full-scale BESs are needed to maximize degradation of organics in the anode and thus increase the electron availability in the cathode. In this sense, a new reactor design proposed by Ran et al. produced hydrogen and methane with 98.0% COD removal rate in the anode [168]. Cheng et al. proposed a new design based on a membraneless BES (rotatable bioelectrochemical contactor) for large scale BES-WWT to recover methane from a synthetic low strength wastewater. This new design achieved significant COD removal and methane yield (404.88 mmol·day^−1^·m^−2^) with a CE over 80% [179]. Katuri et al. evaluated another novel electrochemical bioreactor composed of conductive hollow-fiber membranes used to treat low-organic strength solutions, removing more than 95% of the initial COD (320 mg·L^−1^). Furthermore, up to 71% of the substrate energy was recovered as methane rich biogas at an applied voltage of 0.7 V and a net energy needed of 0.27 kWh m^−3^ of wastewater, far from the 1–2 kWh·m^−3^ currently needed for wastewater treatment using aerobic membrane bioreactors [180]. Besides the microbial oxidant capability of bioelectrodes, the electrode itself might act as a physical filter of solids with removal efficiencies of 83% of suspended solids and 77% of the COD in a study using real wastewater and carbon felt as anodic electrode [175]. However, suspended solids in the electrode might lead to electrode clogging, which makes the reactor dependent on an effective cleaning system.

Moreno et al. assessed the use of a BES to treat low-strength wastewater in the surface of the anode, while recovering methane in the cathode surface with promising results in a 3 L single-chambered reactor [139]. The authors suggested that controlling cathode potential, in parallel with a smart electrode choice with higher specific surface area in both the cathode and the anode, might lead to better results due to higher CO_2_ conversion and larger methanogenic communities attached on the electrode. In this sense, Feng and Song proved, in a similar but smaller setup, that coal tar pitch is a biocompatible binder for the enrichment of electrochemically active bacteria, and nickel is a good catalyst for electron transfer on the anode, which enhanced the methane production in the biocathode [181]. Moreover, studies with very low CE have been carried out in semi-pilot scale tubular BES fed with real domestic wastewater [176].

Nevertheless, the extent to which these challenges can be resolved will eventually determine how bioelectrochemical wastewater treatment can be practically implemented.

Besides the combination of cathodic electromethanogenesis and anodic WWT in BESs, several authors proposed alternative anodic processes such as bioremediation with great success. Huang et al. successfully recovered cobalt with simultaneous methane and acetate production in a biocathode [182]. In this study, Co(II) was added and subsequently reduced in the biocathode, while sodium acetate was oxidized in the bioanode, thus mimicking cathodic bioremediation and anodic WWT. Furthermore, Jiang et al. performed anodic sulfide removal and cathodic electromethanogenesis in a microbial fuel cells-microbial electrolysis cell coupled system [44]. These proofs of concept demonstrate that a low energetic cost methodology for bioremediation through BESs is possible when electromethanogenesis occurs in the biocathode. However, its potential as electron donors to electromethanogenic biocathodes at this early stage of development is questionable. Future research should focus on how to increase the bioremediation potential of those anodic processes, identify the species involved and chose their best operation parameters. In addition, the treatment of different substrates such as wastes from the food industry, agricultural by-products or pure organics has successfully been investigated as anodic substrates [183,184]. Therefore, these processes must be considered as potential electron donors for methanogens colonizing the cathodes.

## 4. Current Limitations in Electromethanogenesis and Proposed Strategies

Although many limitations still need to be addressed to optimize this technology and make it economically feasible. Constraints regarding side reactions, mass transfer, inoculum type, electrode material, anode-cathode separation, operation parameters, system design or scaling-up are currently the bottlenecks of a future technology based on electromethanogenesis.

### 4.1. Side/Parasitic Reactions

Methane and oxygen diffusion to anode and cathode respectively occurs in BES. Gas diffusion through the membrane is considered one of the main limitations lowering the CE of the process [18] due to losses in the methane yield and the role of diffused oxygen as an alternative electron acceptor. Moreover, most of the microorganisms in the cathode chamber are likely to be sensitive to oxygen. Improvements on the reactor design and manufacture of low gas permeability membranes must be achieved to definitely prevent it. Despite this problem is inherent to current available membranes, recent studies have addressed this issue concluding that proton exchange membrane allows a higher methane production [185].

Other electron sinks in the system are reactions of reduced compounds present present in the buffer solution of the cathodic culture medium (e.g., sulfides, Fe^2+^) with oxidants generated in the anode chamber (e.g., O_2_, H_2_, or Cl_2_), because membranes are not completely impermeable to neutral and positively charged small molecules. Some of these molecules could be re-oxidized or re-reduced at the anode or cathode, or react in solution and generate intermediates [48].

Moreover, oxygen diffusion to mixed culture biocathode compartment enhances the growth of methanotrophic species able to use methane as carbon source and thus decreasing its yield [19]. Nonetheless some authors consider the presence of methanotrophs beneficial to preserve anaerobic conditions and thus keeping anaerobic methanogens active [19]. Other factors such as competitive processes and bacterial growth could lead to a reduction in the CE of the process. In addition, the methanotroph community is rather interesting from the commercial point of view since recent studies have suggested the new methanotroph-based biorefinery concept [186]. Methanotrophs are able to generate multiple products from methane fermentation such as feed supplement, ectoine, sucrose, biofuels, biopolymers, surface layers, metal chelating protein, enzymes and/or heterologous proteins [187]. Nowadays, companies such as Mango Materials are commercializing biomethane to bioplastics [128] since a life cycle analysis has recently shown that biomethane conversion to bioplastics is carbon neutral and energetically and economically feasible [188]. Additionally, a methane-oxidizing community may serve as methane scavenger in the exhaust gas resulted from a biogas upgrading process [189].

### 4.2. Mass Transport

Transport rates within the BES system rather limit the electromethanogenesis performance. Concentration gradients at anode and cathode inherent to several fluxes within the system such as electron donor and acceptor, ionic acidity and alkalinity, electron transport at the biofilm, and reactant/product crossover affect its overall performance [190]. However, improved MEC designs focused in overcome transport limitations could result in higher performances and thus better product yields. A more efficient electromethanogenesis process in large-scale systems will be achieved when these transport processes occur at the same rate that microorganisms consume electrical current.

One successful strategy is to combine the catalyst layer and a hydrophobic gas diffusion layer, creating a three-phase interface at the electrode, thus the availability of the substrates to the biocatalyst on the cathode surface is enhanced [191]. Such approach has been proven in the microbial catalysis of CO_2_ reduction to multi-carbon compounds, which makes it also applicable to electromethanogenesis. Moreover, strategies proposed for a successful up-scaling are using thinner electrodes to maintain a 3D structure for biofilm growth and decreasing ohmic losses [192,193], removing ionic membranes or placing anode and cathode close to each other to minimize acidity and alkalinity gradients, as well as treating high strength and high alkalinity wastewaters in the anode to alleviate gradients to the cathode chamber [194].

In addition, limitations between phases due to poor solubility of CO_2_ and H_2_ are far for being avoided through the classic BESs configurations, even though the in situ H_2_ production in the liquid phase of the reactor eases its direct utilization by the microbes. Solubilization profile in fresh water is completely different as compared to salt water, fact that complicates the operation due to oscillations in the system. Moreover, bacteriostatic effect of using CO_2_ as substrate can hamper bacteria growth [195] and thus decrease the overall performance of mixed consortia biofilms [120]. The extremely poor aqueous solubility of H_2_ also compromises mediated electromethanogenesis, which is known to occur in the aqueous phase containing the cathodic methanogenic community. Process operation under H_2_ mass transfer limitation is known to decrease the efficiency of CH_4_ production at the expenses of an enhanced biomass formation [196], resulting in the need to operate at high retention times. The implementation of high-mass-transfer gas phase bioreactors (i.e., two-phase partitioning or Taylor flow) designs to biocathodes may lead to an increase in the volumetric CH_4_ productivities of up to 1 order of magnitude [197], due to a higher H_2_ and CO_2_ bio-availability to planktonic hydrogenotrophic communities.

### 4.3. Inoculum Type

The nature of the electromethanogenic inoculum is of major importance in the productivity of a biocathode. Siegert et al. reported higher methane yields in biocathodes when hydrogenotrophic methanogens where presented in the inoculum [73]. In agreement with other studies [44,45,55], these findings suggest that the presence of Archaea of the hydrogenotrophic genera *Methanobacterium* and *Methanobrevibacter* are crucial to produce methane in BESs and their presence in the inoculum favours electromethanogenesis processes. Obviously methanogens must be included in a wide range of inoculum, but also the presence of effective electron uptake species will ensure a free electron flow from the electrode to electromethanogens unable to attach on the electrode surface.

On the other hand, a study conducted by Pissciota et al. reported the successful transfer of sediment-type microbial fuel cells bioanode suspension as inoculum to sterile cathodes made of graphite plates, carbon rods, or carbon brushes in new BESs [63]. The authors reported an increase of current demand and methane production related to the growth of methanogen *Methanocorpusculum labreanum* in the biocathode. Thus, the versatility of bioanode community to live in a methanogenic biocathode and the ability of methanogens present in the inoculum to grow on a variety of cathode substrates were demonstrated.

### 4.4. Electrode

Electrode material and its surface are key factors that govern electron exchange and methane formation efficiencies in electromethanogenesis. Current efforts to improve performance are focused on combining new and existing materials [20,198], re-shaping them [181,199] or applying different pre-treatments to the electrode surface [200] in order to increase biocompatibility. The use of modified carbon cloth with chitosan or cyanuric chloride, which is relatively inexpensive, showed a 6 to 7-fold increase in microbial electrosynthesis activity [198]. Aryal et al. developed a 3D-network cathode as an effective approach to improve microbe-electrode interactions leading to productive CO_2_-reducing biocathodes [199]. LaBarge et al. confirmed that microbes related to electromethanogenesis and exocellular electron transfer were enriched on a pretreated granular activated carbon, improving methane generation rates and decreasing startup times [200]. The pretreatment consisted of adding such as inorganics (hydrogen gas) or organics (methanol, acetate and propionate) substrates to the inoculum communities, and enriching them in archaea, *Geobacter* spp., and sulfate-reducing bacteria prior to their inoculation.

In addition, Zhen et al. obtained inspiring results when they evaluated the performance of a hybrid biocathode covered with graphite felt [20]. In this study, methane production was highly comparable to that originally reported for Pt-catalysed carbon cloth by Cheng et al. [8], for carbon paper by Villano et al. [13], for graphite/carbon fiber brush by Pisciotta et al. [63] and Siegert et al. [11], for Pt by Siegert et al. [11], and for graphite felt (GF) alone by Van Eerten-Jansen et al. [18]. The use of the hybrid GF-biocathode in these studies showed great promise in promoting electromethanogenesis of BES processes not only because of high energy recoveries but also as a result of the ease of operation with low-cost materials [20]. Moreover, much attention has been focused on successfully improving of electron uptake in CO_2_ reducing biocathodes by modifying the electrode surface. For instance, Jourdin et al. used a macropore of about 0.6 mm in diameter, in a highly open macroporous reticulated vitreous carbon electrodes. This electrode shape seemed optimal to achieve a good balance between total surface area available for biofilm formation and effective mass transfer between the bulk liquid and the electrode and biofilm surface [201].

Nevertheless, the lack of understanding of the mechanisms by which electrons are transferred from cathodes to cells is a clear hurdle for an effective design of cathode materials. Thus, new knowledge about electron transfer mechanisms will definitely lead to the design of new and more effective electrodes and will boost the electromethanogenesis performance. Further research is expected to tune materials and cathode potentials to best interact with the appropriate electron carriers, electromethanogens or efficient electron uptake species functioning as electron providers to electromethanogens. Improve attachment and biofilm growth of electron uptake species might greatly increase areal current densities and improve the performance of electromethanogenic biocathodes.

### 4.5. Anode-Cathode Separation: Membrane

An effective selection of the cathode-anode separation is crucial in electromethanogenic BESs. The type of ion selective membrane not only determines stability and production levels [202], but also the economic feasibility of the technology. Moreover, Babanova et al. assessed a Nafion 117 and an Ultrex CMI-7000 membrane in an electromethanogenic BES, pointing out differences in the electrochemical operation and in the microbial taxonomic composition and dynamics within the biocathode [75]. This study emphasizes the need of a thorough research of membrane types commonly used in electromethanogenic setups to determine and evaluate the impact of its usage in terms of methane production, electrochemical performance, microbial population and dynamics, scalability and practical application.

In addition, Hernandez-Flores et al. determined that agar-containing membranes could replace high cost conventional membranes, thus improving the economic feasibility of electromethanogenesis [203]. The proposed low cost membranes also avoid the use of chemicals needed for the pretreatment (i.e., hydrogen peroxide, sulphuric acid, etc.), which nowadays generates hazardous wastes and increases its cost. For this reasons, a deeper assessment of a broader range of alternative low cost membranes must be carried out.

Therefore, an appropriate membrane selection should be carefully considered in future elelectromethanogenic BES applications and fundamental studies since it may induce significant impacts on system efficiencies, microbial community selection and final costs.

### 4.6. Operation Parameters

The cathode potential is undoubtedly the most important operation parameter, since it drives the hydrogen production and subsequently the methanogenic process in BESs. The combination of cathode potential with the energy losses at the anode, ohmic losses, and transport and pH losses, determines the overall internal resistance [204], which is also a crucial parameter. Moreover, the assessment of the overall performance of electromethanogenesis must be performed in terms of cathodic and energy efficiencies [22].

The assessment of long-term operation studies of electromethanogenic BESs is essential, however only a few have been conducted so far. Van Eerten-jansen et al. experienced internal resistances at long term, ending up in low energy efficiencies. The study showed that cathode and anode losses where dominant in the short term, whereas pH gradient—due to water oxidation in the anode—and transport losses through the membrane were critical in the long term [18].

The reduction of energy losses at the cathode (cathode overpotential) is essential to improve the performance of electromethanogenic-like reactors. For that reason, new strategies on increasing the mass transfer of substrates and products in the electrode or improving the microbial adhesion to the cathode are crucial to overcome such limitation. A forced catholyte flow through the electrode may increase the mass transport of substrates towards the electrode and products away from it [205]. In addition, a better microbial adhesion to the electrode must be in consideration to decrease the overpotential in electromethanogenic systems [11,206,207]. In this sense, promising approaches to increase the microbial adhesion are (i) novel electrode materials and (ii) decrease the electrostatic repulsion of the negatively charged microbial and electrode surfaces by increasing the hydrophobicity of the electrode. Moreover, Luo et al. considered using a microbial reverse-electrodialysis methanogenesis cell in order to decrease the energy requirement to overcome large cathode overpotentials [208]. In this study, the authors successfully minimized the energy by placing a reverse electrodialysis stack between the anode and the biocathode, creating a salinity gradient which was subsequently converted to electrical energy.

### 4.7. System Design and Scaling-Up

Although the results have been less than sterling, many efforts to scale-up BES-based technologies have already been made. In fact, Cusick et al. successfully constructed and tested a pilot-scale (1000 L) continuous flow microbial electrolysis cell for current generation and COD removal with winery wastewater [209]. Techniques for scaling up microbial fuel cells are also used for the upscaling of electromethanogenic-based reactors. Nowadays, the proposed strategies are either a connection of single small cells together, or the increase of a single BES volume. However, usually the process of scaling up requires a change in electrode spacing and orientation, which impacts the internal resistance of the system and therefore decreases the overall performance. On the other hand, doubling the anode and cathode electrode sizes can increase current density, which together with a correct cathodic electrode choice will result in an increase of the methane production rate. Simplifying reactor design by both removing potentiostatic control of the cathode and the membrane separating the anode and cathode is not valid for methanogenesis purposes, which will inhibit methanogenesis due to the production of oxygen.

The optimization of electrochemical, biological, hydraulic and mass transport requirements should be performed as a result of merging existing configurations of industrial and electrochemical reactors [120]. Roy et al. proposed key aspects for the configuration and the engineering of electromethanogenic-like reactors such us (i) maximize microbial cell retention, keeping in mind requirements of substrates and target products; (ii) favor electron transport; and (iii) use separators between anode and cathode for a simultaneous product recovery [120]. In regards to cell retention, planktonic microbial culture must be retained, to improve configurations and performance. Cell retention can be achieved by recycling and using membrane reactors. In addition, Blanchet et al. confirmed that hydrogen produced on the cathode by water electrolysis is an essential mediator in the microbial electrochemical reduction of CO_2_, underlying the importance of the hydrogen route in up-scaling electrosynthesis for microbial CO_2_ reduction [210].

The use of low-cost membranes and cloth electrodes are always desirable when designing large scale reactors. However, low cost clothes may slowly get degraded in large scale systems by the microorganisms, which might reduce the long-term stability of the process. In this sense, non-biodegradable low-cost separators with high proton transfer rates are suggested. Developments in the field of polymer electrolyte membranes applied to fuel cell stacks would be very valuable to avoid constraints when designing electromethanogenesis reactors.

Although those restrictions still compromise larger electromethanogenic prototypes, companies such as Electrochaea (available online: http://www.electrochaea.com) or Cambrian Innovation (available online: http://cambrianinnovation.com/resources/) are currently developing real-scale reactors, which highlight the existence of a market niche for electromethanogenesis-based technology. In fact, many patents have been registered so far [30,211,212,213,214] in view of upscaling the technology. Nevertheless, large scale undertakings are crucial not only to obtain know—how of scaling-up electromethanogenesis, but also to perform techno-economic analyses. Both Electrochaea and Cambrian Innovation initiatives are able to deal with gas streams with variable composition (e.g., biogas), a key aspect to develop a robust technology in the future.

There is an increasing need for innovative prototypes at the industrial level, especially those able to keep high methane yields regardless of operation parameters when performing electromethanogenesis.

## 5. Outlook

The broad range of applications for electromethanogenesis opens new technological niches to be fulfilled. Its nature of one-step process, capability to perform at ambient temperature and the low-cost inputs—CO_2_ as the sole carbon source—make nowadays electromethanogenesis a promising technology. However, its success will be closely subjected to new insights provided by basic research, which will help to overcome the limitations pointed out throughout this review.

Additional studies dealing with the elucidation of different methane production mechanisms, together with conclusive roles of the intermediates in methanogens, will lead to an optimization of the biocathode for methane formation. In this sense, a deeper understanding of electron uptake mechanisms utilized by electroactive species and their identities is needed. For that reason, further research with defined co-cultures that would provide new knowledge of interspecific interactions may represent a foundation for an engineering strategy for electromethanogenesis and provide a means to understand complex cathodic biofilms. The increase of the methane yield and energy efficiencies of the electromethanogenic processes will undoubtedly place the technology in a privileged position to compete with well-established technologies. Furthermore, the future up-scaling of the process should successfully address the maintenance of high production rates regardless of possible trace contaminants in real carbon dioxide streams.

Electromethanogenic BES should take advantage of the rapid development in digester designs adapted to novel fermentation processes. There is an increasing necessity to adapt new engineered designs to electromethanogenic reactors that will solve current constraints dealing with physicochemical limitations. Moreover, ongoing advances in new electrode materials and shapes should also harness the birth of new electromethanogenic reactors with better microbe adhesion properties. Those improvements will lead to a better interaction between electron-accepting microorganisms involved in electromethanogenesis and the cathode surface, therefore increasing methane yields.

Moreover, the raising of an environmental awareness concerning global warming has opened an invigorating scenario for the development of novel green technologies. Governments worldwide are willing to support initiatives that aim to mitigate climate change, and thus electromethanogenic-based technologies are placed in a privilege position.

Electromethanogenesis has born to be an alternative to current power-to-gas processes such as the Sabatier process or AD, despite the uncertainties about whether it will work as a stand-alone system or a supplementary system.

The authors are optimistic in the feasibility of electromethanogenesis to be the basis of robust, feasible and marketable technologies in the short term. Such upcoming technologies are expected to play an important role in the near future of global bioenergy field, placing them on the cutting edge of the foreseen biofuel production scenario.

## Figures and Tables

**Table 1:** Overview of the reaction equations that could occur in the possible electron transfer mechanisms of bioelectrochemical methane production. Based on reactions described in Van Eerten-jansen et al. 2014 [17]. Additionally, microorganisms described to possibly take part in the electromethanogenesis process are related to each reaction.

Reaction/Process	Type (Place)	References in Figure ^3^	Microorganism [References]
${\mathrm{HCO}}_{3}^{-}+{9H}^{+}+{8e}^{-}\overset{\mathrm{electricity}}{\to }{\mathrm{CH}}_{4}+{3H}_{2}O$	BEC (C)	[1]	*Methanobacterium palustre* [8,19]
			*Methanococcus maripaludis* [48,49,50]
			*Methanobacterium-like (IM1)* [51,52]
			*Methanosaeta* spp. [34]
			*Methanosaeta concilii* [33]
			*Methanosarcina barkeri* [12,35]
			*Methanothermobacter thermautotrophicus* [12,53]
			*Methanosaeta harundinacea* [34]
			*Methanothermobacter* sp. [54]
			*Methanoculleus* sp. [54]
			*Methanobacterium* sp. [14,54,55]
			*Methanosarcina mazei* [56]
			*Methanothermobacter-like* [15]
			*Methanobacteriaceae* [20]
			*Methanobacterium petrolearium* [33]
			*Methanobacterium subterraneum* [33]
			*Methanothermobacter thermautotrophicus* [9]
			*Methanosaeta concilii* ^2^ [34]
${2H}^{+}+{2e}^{-}\overset{\mathrm{electricity}}{\to }H_{2}$	BEC (C)	[2,3]	*Desulfovibrio vulgaris* [57,58]
			*Geobacter sulfurreducens* [12,59,60]
			*Pelobacter carbinolicus* [35]
			*Hydrogenophaga caeni* (EMB71) [19]
			*Desulfovibrio putealis* (B7-43) [19]
			*Desulfovibrio paquesii* [61]
			*Firmicutes* [14,62]
			*Proteobacteria* [62]
			*Bacteroidetes* [62]
			*Actinobacteria* [62]
			*Rhodococcus* sp. [63]
			*Sphingobacteriales* [55]
			*Desulfovibrio* spp. [63]
${2\mathrm{HCO}}_{3}^{-}+{9H}^{+}+{8e}^{-}\overset{\mathrm{electricity}}{\to }{\mathrm{CH}}_{3}{\mathrm{COO}}^{-}+{4H}_{2}O$	BEC (C)	[4]	*Sporomusa ovata* [64]
			*Sporomusa sphaeroides* [65,66]
			*Sporomusa silvacetica* [65]
			*Clostridium aceticum* [65]
			*Clostridium ljungdahlii* [65]
			*Moorella thermoacetica* [65]
			*Clostridium thermoaceticum* [67]
			*Acetobacterium* spp. [42,55,68,69,70]
${\mathrm{HCO}}_{3}^{-}+{2H}^{+}+{2e}^{-}\overset{\mathrm{electricity}}{\to }{\mathrm{HCOO}}^{-}+H_{2}O$	BEC (C)	[5]	*Moorella thermoacetica* [71]
			*Clostridium formicoaceticum* [71]
${\mathrm{HCO}}_{3}^{-}+H_{2}+H^{+}\to {\mathrm{CH}}_{4}+{3H}_{2}O$	BC (C)	[6]	*Methanobacterium* sp. [14,43,55,72]
			*Methanobacterium palustre* [19]
			*Methanobacterium aarhusense* [19]
			*Methanobacterium formicicum* [72]
			*Methanobrevibacter arboriphilus* [44,56]
			*Methanocorpusculum parvum* [44]
			*Methanocorpusculum labreanum* [63]
			*Methanobrevibacter* [45,70,73]
			*Methanosarcina* sp. [43]
			*Methanosarcina mazei* [56]
			*Methanoculleus* sp. [43,74]
			*Methanomicrobiales* [20]
			*Methanobacterium petrolearium* [33]
			*Methanobacterium subterraneum* [33]
			*Methanothermobacter thermautotrophicus* [9]
			*Methanothermobacter* sp. [74]
			*Methanococcus maripaludis* [47]
${2\mathrm{HCO}}_{3}^{-}+H_{2}+H^{+}\to {\mathrm{CH}}_{3}{\mathrm{COO}}^{-}+{4H}_{2}O$	BC (C)	[7]	*Acetobacterium woodii* [65]
			*Sporomusa silvacetica* [65]
			*Clostridium aceticum* [65]
			*Clostridium ljungdahlii* [65]
			*Moorella thermoacetica* [65]
			*Clostridium* sp. [75]
${\mathrm{CH}}_{3}{\mathrm{COO}}^{-}+H_{2}O\to {\mathrm{CH}}_{4}+{\mathrm{HCO}}_{3}^{-}$	BC (C)	[8]	*Methanosaeta* sp. [45,73,76]
			*Methanosarcina* sp. [7,74,77]
			*Methanosarcina thermophila* [72]
			*Methanosaeta harundinacea* [34]
			*Methanosarcina mazei* [38,56]
${\mathrm{HCO}}_{3}^{-}+H_{2}\to {\mathrm{HCOO}}^{-}+H_{2}O$	BC (C)	[9]	*Acetobacterium woodii* [78]
			*Candida boidinii* [78]
${\mathrm{HCOO}}^{-}+{3H}_{2}+H^{+}\to {\mathrm{CH}}_{4}+{2H}_{2}O$	BC (C)	[10]	*Methanococcus maripaludis* [79]
			*Methanomicrobiales* [20]
			*Methanobacterium formicicum* [72]
${\mathrm{CH}}_{4}+{2O}_{2}\to {\mathrm{CO}}_{2}+{2H}_{2}O$	BC (C)	n.s.	*Acidovorax caeni (R-24608)* [19]
			*Hydrogenophaga caeni (EMB71)* [19]
			*Methylocystis sp. (SC2)* [19]
Unknown ^1^	-	-	*δ-Proteobacteria* [14,45]
			*Geobacter* sp. [54,76,80]
			*Pelobacter carbinolicus* [35]
			*Desulfovibrio* spp. [75,81]
			*Synergistetes-like* [15,72]
			*Thermotogae-like* [15]
			*Methylocystis* sp. [14]
Unknown ^2^	-	-	*Methanospirillum hungatei* [12]
			*Methanoregula boonei* [12]
			*Methanocopusculum bavaricum* [12]
			*Thermoplasma* sp. [12]
			*Methanoculleus bourgensis* [72]
Unknown ^3^	-	-	*Methanobacterium sp. (YCM1)* [38]
			*Methanobacterium bryantii (RiH2)* [38]
			*Methanosarcina mazei (Tuc01)* [38]
			*Methanosarcina thermophila* [72]
			*Methanobacterium arcticum (M2)* [75]
			*Methanobacterium bryantii (MOH)* [75]
${4H}_{2}O\to {2O}_{2}+{8H}^{+}+{8e}^{-}$	Ch (A)	n.s.	

^1^ Higher relative abundance correlated with higher CH_4_ production in electromethanogenic biocathode; ^2^ Associated with syntrophic associations with methanogens in the biocathode to produce CH_4_; ^3^ Archaea identified in electromethanogenic BES reactor biocathodes; BEC: Bioelectrochemical; BC: Biochemical; Ch: Chemical; C: Cathode; A: Anode; n.s.: Not shown.

**Table 2:** Overview of the operational parameters and performance of methane-producing biocathodes.

Reactor Operation	Cathode Material	Cathode Potential (V vs. SHE)	Cathode Working Volume (mL)	Cathode Specific Surface (cm^2^)	Anode Reaction	Current Density (A·m^−2^) ^5^	CH_4_ Yield (mmol·day^−1^·m^−2^)	CE (%)	Reference
B	Carbon paper	−0.90	150	8	WO	0.69 (^6^)	400	80	[13]
B	Carbon black powder + Pt ^1^	<−0.55	100	9.28	n.r.	n.r.	35.85 (^2,3^)	>100	[11]
B	Graphite rod	<−0.4	350	13	n.r.	0.05 (^8,9^)	3.5	80	[52]
B	Graphite granules	−0.8	420	5700	WO	0.07 (^10^)	5.1	75	[14]
B	Carbon paper	−1.0 (^4^)	10	3	n.r.	3.00 (^8^)	87.9	19	[53]
B	Carbon paper coated + carbon layer	−1.0 (^4^)	10	3	n.r.	n.r.	95.5	96	[9]
B	Graphite plate	−0.7	200	64.5	BO	1.00 (^9^)	48.05 (^2,3^)	83	[33]
B	Carbon felt	−0.6/−0.7	240	98	SO + BO	n.r.	29.26 (^2,3^)	51	[44]
B	Granular graphite	−0.59	75	n.r.	n.r.	n.r.	n.r.	55	[55]
B	Carbon felt	−0.95	240	49	n.r.	n.r.	1062 (^2,3^)	56.7	[137]
B	Carbon felt	−1.25 (^4^)	n.r.	42	BO	n.r.	>400	>95	[12]
B	Graphite felt	−1.5 (^4^)	40	4700	WO	n.r.	n.r.	n.r.	[27]
B	Graphite fiber brush	−0.439	120	13.8	WO	0.04	63.48 (^3^)	n.r.	[63]
B	Graphite bar	−0.5	100	8	n.r.	n.r.	0.22	n.r.	[47]
C	Graphite felt	<−0.55	n.r.	250	HO + WO	0.21 (^7,10^)	22.2	23	[18]
C	Graphite granules	−0.93	860	11,094	BO	0.10 (^8,10^)	8.84 (^3^)	79	[138,139]
C	Graphite granules	−0.8	420	5700	WO	0.20 (^10^)	15.4	69	[14]
C	Graphite plate	−0.7	800	64.5	BO	>3 (^9^)	155 (^2,3^)	>80	[33]
C	Graphite felt	−0.7	240	250	WO	2.90 (^7,10^)	477.7 (^2,3^)	<60	[17]
C	Carbon cloth	−0.5	110	85.5	n.r.	0.04 (^10^)	0.58 (^2,3^)	63	[38]
FB	Graphite plate	−0.6	250	4150	WO	n.r.	1901.19 (^2,3^)	n.r.	[74]
FB	Graphite granules	−0.85	860	11,094	BO	0.02 (^9^)	1.58 (^3^)	74	[140]
FB	Graphite fiber brush	<−0.5	1750	947	BO	0.30 (^8,10^)	200	96	[8]
FB	Graphite felt	<−0.6	620	290	HO + WO	1.60 (^7,10^)	205 (^3^)	99	[19]
FB	Carbon cloth	0.8 (^4^)	150	80	BO	0.17 (^7,9^)	1103	>90	[15]
FB	Plain carbon felt	−0.75 (^4^)	110	40	BO	2.60 (^8,9^)	386	98	[141]
FB	Carbon stick	−0.7	400	11	WO	n.r.	397 (^3^)	24.2	[10]
FB	Carbon stick + graphite felt ^1^	−1.2	200	22	WO	n.r.	2911.99 (^3^)	194.4	[20]

Studies assessed were not conducted with the enhancement of methane production as main purpose. CO_2_ was not the only carbon source in some of them. ^1^ Different cathode materials were studied in this work; ^2^ Highest production rate reported; ^3^ Calculated from the data reported (temperature, pressure, cathode surface area, etc.); ^4^ Applied cell voltage (cathode potential not reported); ^5^ Calculated at standard temperature and pressure (STP, 298.15 K and 1 bar); ^6^ Calculated from an average of a range reported in the study; ^7^ Projected surface area: projected 2D active area of the 3D cathode; ^8^ Geometric surface area: total outer active surface area of the cathode, not including pores; ^9^ Study states the maximum value; ^10^ Study states the average value; B: Batch; C: Continuous; FB: Fed-batch (medium in the cathode compartment substituted with fresh medium when considered); BO: Biotic organic oxidation; WS: Water oxidation; SO: Sulfide oxidation; HO: hexacyanoferrate (II) oxidation; n.r.: not reported.

## Abbreviations

AD	Anaerobic digestion
BES	Bioelectrochemical system
CCE	Cathode capture efficiency
CE	Coulombic efficiency
COD	Chemical oxygen demand
IET	Interspecies electron transfer
GF	Graphite felt
SHE	Standard hydrogen electrode
WWT	Wastewater treatment
